# Effects of Treadmill Running at Different Light Cycles in Mice with Metabolic Disorders

**DOI:** 10.3390/ijms242015132

**Published:** 2023-10-13

**Authors:** Anna Nikolaevna Zakharova, Kseniya Gennadievna Milovanova, Anna Alekseevna Orlova, Elena Yuryevna Dyakova, Julia Gennadievna Kalinnikova, Olesya Vadimovna Kollantay, Igor Yurievich Shuvalov, Alexander Valerievich Chibalin, Leonid Vladimirovich Kapilevich

**Affiliations:** 1 Department of Sport Tourism, Sport Physiology and Medicine, National Research Tomsk State University, 634050 Tomsk, Russia; naffys@mail.ru (K.G.M.); anna.orlova.96@mail.ru (A.A.O.); adyakova@yandex.ru (E.Y.D.); kisa101090@yandex.ru (J.G.K.); olesya.tay@mail.ru (O.V.K.); oleg-100500-lol@mail.ru (I.Y.S.); alexander.chibalin@ki.se (A.V.C.); kapil@yandex.ru (L.V.K.); 2 Department of Molecular Medicine and Surgery, Section of Integrative Physiology, Karolinska Institutet, 17177 Stockholm, Sweden; 3 Central Research Laboratory, Siberian State Medical University, 634050 Tomsk, Russia

**Keywords:** mice, skeletal muscles, running load, metabolic disorders, obesity

## Abstract

Type 2 diabetes mellitus accounts for about 90% of cases of diabetes and is considered one of the most important problems of our time. Despite a significant number of studies on glucose metabolism, the molecular mechanisms of its regulation in health and disease remain insufficiently studied. That is why non-drug treatment of metabolic disorders is of great relevance, including physical activity. Metabolic changes under the influence of physical activity are very complex and are still difficult to understand. This study aims to deepen the understanding of the effect of physical exercise on metabolic changes in mice with diabetes mellitus. We studied the effect of forced treadmill running on body weight and metabolic parameters in mice with metabolic disorders. We developed a high-fat-diet-induced diabetic model of metabolic disorders. We exposed mice to forced treadmill running for 4 weeks. We determined glucose and insulin levels in the blood plasma biochemically and analyzed Glut-4 and citrate synthase in M. gastrocnemius muscle tissue using Western blotting. The research results show that daily treadmill running has different effects on different age groups of mice with metabolic disorders. In young-age animals, forced running has a more pronounced effect on body weight. At week 12, young obese mice had a 17% decrease in body weight. Body weight did not change in old mice. Moreover, at weeks 14 and 16, the decrease in body weight was more significant in the young mice (by 17%) compared to the old mice (by 6%) (*p* < 0.05). In older animals, it influences the rate of glucose uptake. At 60 min, the blood glucose in the exercised older mice decreased to 14.46 mmol/L, while the glucose concentration in the non-exercised group remained at 17 mmol/L. By 120 min, in mice subjected to exercise, the blood glucose approached the initial value (6.92 mmol/L) and amounted to 8.35 mmol/L. In the non-exercised group, this difference was 45%. The effects of physical activity depend on the time of day. The greater effect is observed when performing shift training or exercise during the time when animals are passive (light phase). In young mice, light phase training had a significant effect on increasing the content of Glut-4 in muscle tissue (84.3 ± 11.3%, *p* < 0.05 with control group—59.3 ± 7.8%). In aged mice, shift training caused an increase in the level of Glut-4 in muscle tissue (71.3 ± 4.1%, *p* < 0.05 with control group—56.4 ± 10,9%). In the group of aged mice, a lower CS level was noticed in all groups in comparison with young mice. It should also be noted that we observed that CS increased during exercise in the group of young mice, especially during light phase training. The CS content in the light phase subgroup (135.8 ± 7.0%) was higher than in the dark phase subgroup (113.3 ± 7.7%) (*p* = 0.0006). The CS decreased in aged chow-fed mice and increased in the high-fat-fed group. The CS content in the chow diet group (58.2 ± 5.0%) was 38% lower than in the HFD group (94.9 ± 8.8%).

## 1. Introduction

Type 2 diabetes mellitus (T2DM) accounts for about 90% of cases of diabetes and is considered one of the most important problems of our time. Its pathogenesis is associated with insulin resistance in peripheral tissues and, as a result, an increase in blood glucose concentration [1,2,3]. Despite a significant number of studies of glucose metabolism, molecular mechanisms of its regulation in health and disease remain insufficiently studied [4]. Therefore, of great relevance are non-drug interventions for metabolic disorders, including physical activity. Exercise-induced metabolism is a very complex process. It comprises integrative and adaptive reactions in several tissues and organs at the cellular and systemic levels [5].

We developed a high-fat-diet-induced diabetic model. Such models are thought to be more similar to the disease in humans, taking into account that it is usually caused by dietary manipulation rather than cytotoxic substances. A high-fat diet can lead to obesity, hyperinsulinemia, and impaired glucose homeostasis due to insufficient compensation from the islets of Langerhans [6].

Physical loads of different intensity launch a large number of biochemical, molecular, genetic, and epigenetic mechanisms that underlie the body’s adaptive responses to physiological stress [7]. Physical activity has both a direct effect on the skeletal muscles and a systemic effect on the body. A large number of studies confirm that exercise prevents many diseases and maintains the functioning of the body’s systems at the proper level. In particular, physical activity has a positive effect on metabolic disorders [8,9]. Animal experiments have proven that exercise increases insulin sensitivity and improves glucose tolerance induced by a high-fat diet not only in the studied animals but also in their offspring [10].

At the same time, a number of aspects of the impact of physical activity on metabolism are still incompletely understood. In particular, the age aspect of this problem is of interest. Type 2 diabetes is a disease that develops more often in adulthood. However, most experiments are conducted on young animals [11,12,13,14]. Few studies have addressed the issue in aged mice [15,16].

The prevalence of diabetes increases with age. Older adults with diabetes have a higher risk for hypoglycemia due to altered adaptive physiologic responses. Patients also have such comorbidities as cognitive and functional impairment that interfere with rapid detection and appropriate treatment of hypoglycemia [17].

There is evidence that type 2 diabetes occurs more often in middle-aged and older men who were overweight in early adulthood. According to some sources, on average, diabetes develops at the age of 40–60 years. The average age of the disease is 56 years in men and 60 years in women. However, it should be noted that metabolic disorders develop long before the onset of the disease itself, often before the age of 30 years [18,19].

Physical exercise is a preventive measure for metabolic disorders and diabetes. However, the optimal timing of exercise to maximize health benefits is still largely unknown. Several studies have shown that exercise shows a circadian rhythm. Circadian rhythms have a significant effect on exercise performance. It was found that the time of day in which exercise is performed affects coagulation and fibrinolysis [20]. Morning exercise causes the least damage to hemostasis [21]. Exercise may also influence blood pressure depending on the timing of the day [22]. Moreover, circadian disruption is one of the causes of insulin resistance [23]. It was found that glucose uptake and insulin sensitivity in muscles show circadian rhythms [24]. Exercise performed at different times of the day affects skeletal muscle metabolism and endurance [25] and the daily rhythm of energy expenditure [26,27]. Recent studies in mice showed that exercise induces circadian rhythm remodeling by influencing energy metabolism, use of alternative energy sources, and adaptation of the energy expenditure system [25,28].

Currently, it is known that physical activity affects the content of Glut-4 in muscle cells. G. Lynis Dohm examined changes in Glut-4 in response to different types of physical exercise and diet (high-carbohydrate, high-fat, etc.) in different types of muscle fibers [29].

In rats on a high-fat diet, Glut-4 levels in soleus type 1 fibers and type 2 extensor digitorum longus fibers tended to decrease. Physical activity lowered the level of Glut-4 in both muscles [30].

The effect of training at different times of the day on the level of Glut-4 is insufficiently studied. Taking into account the fact that the level of glucose uptake can change when exercising at different times of the day, it seems relevant to consider the effect of circadian rhythms on Glut-4 production.

Although some research has demonstrated that prolonged physical activity does not affect the expression of the Glut-4 gene in the middle-aged [31,32], there is insufficient evidence of what other age-related effects exercise has on Glut-4 production.

In this regard, it is important to study the Glut-4 production and glucose metabolism in muscle cells in different age periods.

Exercise is an important factor in the prevention and treatment of metabolic disorders [26]. However, the most effective time of day to achieve beneficial health effects remains unknown. There are studies showing that afternoon exercise is more effective than morning exercise at improving blood glucose levels in men with type 2 diabetes [33]. Morning exercise had an adverse effect and increased blood glucose. Repeated further studies of longer training regimens are needed to establish the persistence of this adverse effect. These data highlight the importance of optimizing exercise timing when prescribing exercise for the treatment of type 2 diabetes [33]. Understanding the influence of circadian clock systems on human physiology and its regulation by exercise will provide us with new insights into the treatment of metabolic disorders [34,35]. 

Therefore, our research aimed to study the effect of forced physical exercise on body weight and metabolic parameters in mice with metabolic disorders, taking into account age and circadian aspects.

## 2. Results

### Body Weight in Young Mice

**Figure 1:**
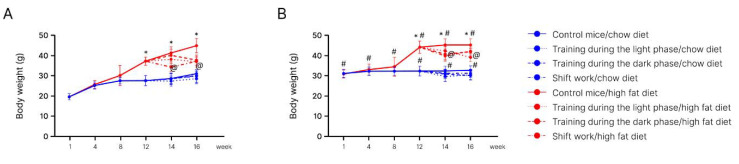
Mice body weight of the experimental and control groups. Panel (**A**)—young mice (age 20 weeks); Panel (**B**)—old mice (age 48 weeks). *—Significantly different (*p* < 0.05) from chow diet. #—Significantly different (*p* < 0.05) from young mice. @—Significantly different (*p* < 0.05) from control.

At the beginning of the experiment, no differences were found between the groups. From week 4, the body weight started to increase. However, there were no significant differences between the high-fat-fed group and the chow-fed group (Figure 1A).

At week 8, the body weight significantly increased in the high-fat-fed mice (35.5 ± 2.0 g) compared to the chow-fed mice (27.8 ± 1.0 g) (*p* < 0.05). It can be argued that a high-fat diet led to the development of obesity in mice after 8 weeks.

From week 12, when the control and experimental groups were divided into four subgroups, we measured the impact of physical activity at different times of the day (light phase, dark phase, and shift training). Significant differences (*p* < 0.05) between the high-fat-fed group (37.5 ± 2.3 g) and the chow-fed group (27.7 ± 1.1 g) remained.

One week after the implementation of the exercise model in the high-fat-fed group, significant differences (*p* < 0.05) were observed in all three subgroups exposed to exercise. The most remarkable difference was observed in the shift training subgroup: the body weight in this group was significantly lower than in other subgroups: 34.6 ± 1.6 g.

At week 13, the body weight of the light phase subgroup in the chow-fed group was significantly different (*p* < 0.05) in comparison with the control group: 27.0 ± 0.7 g and 28.8 ± 0.7 g, respectively. Significant differences (*p* < 0.05) in the body weight of the high-fat-fed group (41.5 ± 1.2 g) and the chow-fed group (28.8 ± 0.7 g) remained.

**Figure 2:**
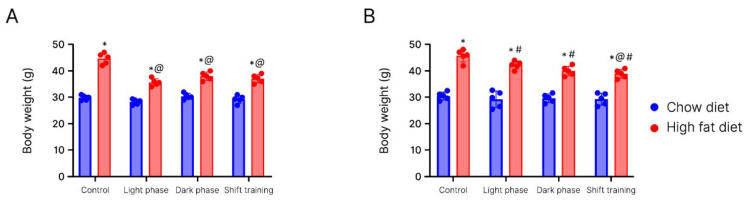
Mice body weight in the experimental and control groups at week 16. Panel (**A**)—young mice (age 20 weeks); Panel (**B**)—old mice (age 48 weeks). *—Significantly different (*p* < 0.05) from chow diet. #—Significantly different (*p* < 0.05) from young mice. @—Significantly different (*p* < 0.05) from control.

At week 16 (final) of the experiment, we observed that high-fat-fed mice (45.2 ± 1.1 g) had a body weight index 1.5 times higher than the chow-fed group (31.0 ±0.9 g) (*p* < 0.001) (Figure 2A). In the high-fat diet group, the body weight differed in all three subgroups exposed to physical activity (*p* < 0.05).

In the chow diet group, the body weight differences were observed only in two subgroups (*p* < 0.05): the subgroup with shift training and the light phase subgroup. In the subgroups with evening exercise, no significant differences were found in comparison with the control group. 

According to the results of the experiment, we discovered that the high-fat diet caused the development of obesity in a group of young-age mice. The body weight in the group of mice on the high-fat diet was significantly higher than in the chow-fed group. The exercise model used in young-age HFD mice demonstrated that exercise at different times of the day decreased the body weight in all groups. The body weight decreased in all subgroups of the chow-fed mice exposed to exercise.

Body weight in old mice. At the beginning of the experiment, no differences were found between the groups. Week 4 saw a significant increase in the body weight (*p* < 0.05). The mean body weight of the high-fat diet group was 35.2 ± 2.0 g, while that of the chow diet group was 32.7 ± 1.3 g (Figure 1B).

At week 8, we observed that the body weight significantly increased in the high-fat diet group (39.3 ± 3.4 g) compared to the chow diet group (32.8 ± 1.3 g) (*p* < 0.05). These differences suggest that a high-fat diet leads to the development of obesity in mice.

From week 12, both groups were divided into four subgroups. One group was not exposed to treadmill running. The other three subgroups exercised at different times of the day. We monitored the effects of exercising during the light phase, dark phase, and shift training.

At week 12, the body weight parameters remained significantly different (*p* < 0.05) between the high-fat diet group (44.3 ± 2.6 g) and the chow diet group (32.2 ± 1.2 g).

At week 13, we observed some differences (*p* < 0.05) in two subgroups in the chow-fed group. The body weights in the light phase subgroup (29.9 ± 1.8 g) and the dark phase subgroup (30.9 ± 0.8 g) were significantly lower in comparison with the control group (32.5 ± 0.8 g).

At week 13, all three high-fat-fed subgroups exposed to physical activity demonstrated differences in body weight (*p* < 0.05). The body weight was 1.2 times lower in the light phase subgroup (40.4 ± 3.0 g) and dark phase subgroup (41.0 ± 4.7 g) than in the control group. 

Significant differences (*p* < 0.05) between the high-fat diet group (45.6 ± 1.3 g) and the chow diet group (32.5 ± 0.8 g) remained.

At week 16 (final) of the experiment, we noticed that the differences in the body weight remained significant (*p* < 0.05) in the groups of high-fat-fed mice (45.6 ± 4.5 g) and chow-fed mice (32.8 ± 2.4 g) (Figure 2B).

In the HFD group, we observed significant differences (*p* < 0.05) in the body weight in all three subgroups exposed to physical activity. Shift training was the most effective. In this group, the body weight was 1.2 times lower (39.2 ± 4.4 g) than in the control group.

Thus, the experiment results demonstrated that the high-fat diet causes the development of obesity in old mice. Body weight in the high-fat-fed group of mice was significantly higher than in the chow diet group. Using a model of physical activity at different times of the day in old mice on a high-fat diet, it was found that shift training is more effective, as it causes a significant decrease in body weight. 

It is important to note certain differences in the effects of physical activity at different times of the day. In young mice, the effect of the shift training appeared faster. A significant decrease in body weight was noticed after two weeks of forced treadmill running. Exercise in the light and dark phases led to a decrease in body weight later. The body weight reached the same values as in the shift training only after 4 weeks.

In old mice, the results were different. After two weeks of forced treadmill running, body weight decreased in all subgroups. However, in the subgroups training during the dark or light phase, weight loss stopped at this point, whereas in the subgroup with the shift training, body weight continued to decrease until week 16. As a result, it turned out to be the lowest value among all the subgroups.

It should also be noted that in young obese mice subjected to running load, the decrease in body weight was more significant compared to aged mice. At week 12, young obese mice had a 17% decrease in body weight. Body weight did not change in old mice. Moreover, at weeks 14 and 16, the decrease in body weight was more significant in the young mice (by 17%) compared to the old mice (by 6%) (*p* < 0.05).

**Figure 3:**
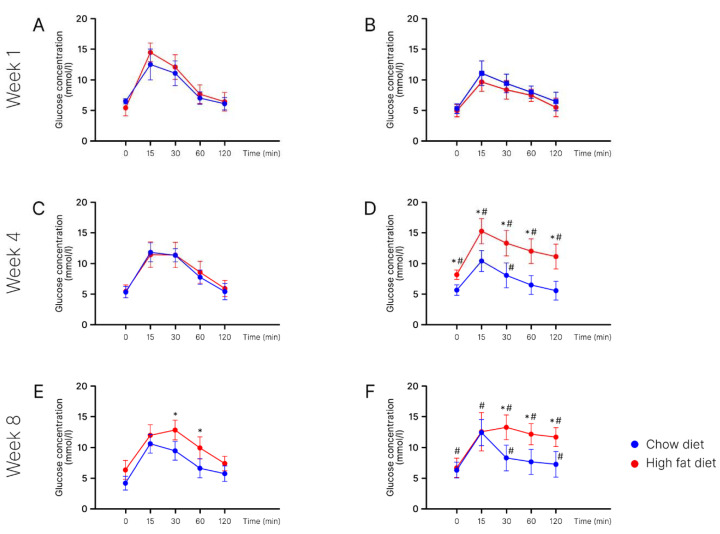
Glucose tolerance test (mmol/L). (**A**,**C**,**E**)—young-age mice; (**B**,**D**,**F**)—old-age mice. *—Significantly different (*p* < 0.05) from chow diet. #—Significantly different (*p* < 0.05) from young mice.

Glucose tolerance test results. GTT results at week 1 of the experiment. At 15 min after the glucose injection, we observed the significant differences between the groups of young (14.4 ± 1.5 mmol/L) and old (9.7 ± 1.5 mmol/L) mice on the high-fat diet (Figure 3A,B).

At 30 min after the glucose injection, we observed the significant differences between the groups of young (12.0 ± 2.0 mmol/L) and old (8.4 ± 1.5 mmol/L) mice on the high-fat diet. At 60 min after the glucose injection, there were significant differences between the groups of young (7.6 ± 1.5 mmol/L) and old (7.5 ± 1.0 mmol/L) mice on the high-fat diet. At 120 min after the glucose injection, significant differences were observed between the groups of young (6.4 ± 1.5 mmol/L) and old (5.5 ± 1.5 mmol/L) mice on the high-fat diet.

GTT results at week 4 of the experiment. Before the glucose injection, there were significant differences in the groups of old mice on the chow diet (5.7 ± 0.9 mmol/L) and high-fat diet (8.2 ± 0.8 mmol/L). The differences were also observed between the groups of young (5.4 ± 1.0 mmol/L) and old (8.2 ± 0.8 mmol/L) mice on the high-fat diet (Figure 3C,D).

At 15 min after the glucose injection, significant differences were observed in the groups of old mice on the chow diet (10.4 ± 1.7 mmol/L) and high-fat diet (15.2 ± 2.0 mmol/L). Moreover, at this time, there were differences between the groups of young (11.3 ± 2.1 mmol/L) and old (15.2 ± 2.0 mmol/L) mice on the high-fat diet.

At 30 min after glucose injection, there were significant differences between the groups of young (11.2 ± 1.1 mmol/L) and old (8.1 ± 2.0 mmol/L) mice on the chow diet as well as between young (11.3 ± 2.1 mmol/L) and old (13.3 ± 2.1 mmol/L) mice on the high-fat diet. At this time, significant differences were found in the groups of old mice on the chow diet (8.1 ± 2.0 mmol/L) and high-fat diet (13.3 ± 2.1 mmol/L).

At 60 min, we saw the significant differences between the groups of young (8.5 ± 1.8 mmol/L) and old (12.0 ± 2.0 mmol/L) high-fat-fed mice. In the group of old mice, there were differences between the chow-fed (6.5 ± 1.5 mmol/L) and high-fat-fed mice (12.0 ± 2.0 mmol/L).

At 120 min, significant differences were observed between the groups of young (5.8 ± 1.3 mmol/L) and old (11.1 ± 2.0 mmol/L) mice on the high-fat diet. In the group of old mice, there were differences between the chow-fed (5.6 ± 1.5 mmol/L) and high-fat-fed ones (11.1 ± 2.0 mmol/L).

GTT results at week 8 of the experiment. Before the glucose injection, we observed the significant differences between young (4.3 ± 1.5 mmol/L) and old (6.3 ± 1.5 mmol/L) mice on the chow diet (Figure 3E,F).

At 15 min after glucose injection, there were differences between young (12.0 ± 1.7 mmol/L) and old (12.5 ± 3.1 mmol/L) mice on the high-fat diet.

At 30 min, there were differences in the groups of young mice on the chow diet (9.4 ± 1.6 mmol/L) and HFD (12.8 ± 1.6 mmol/L). The differences were also observed in the group of old mice on the chow diet (8.3 ± 2.0 mmol/L) and HFD (13.2 ± 2.0 mmol/L). The differences were observed between young and old mice in both the chow-fed group and high-fat-fed group.

At 60 min, differences were observed in the group of young (9.9 ± 1.8 mmol/L) and old (12.1 ± 1.7 mmol/L) mice on the high-fat diet. We noticed differences in the groups of young mice on the chow diet (6.7 ± 1.8 mmol/L) and HFD (9.9 ± 1.8 mmol/L). Differences were also found in the groups of old mice on the chow diet (7.7 ± 1.7 mmol/L) and HFD (12.1 ± 1.7 mmol/L).

At 120 min, there were significant differences between the groups of young (7.4 ± 1.2 mmol/L) and old (11.7 ± 1.5 mmol/L) mice on the high-fat diet. The differences were also observed in the chow diet group between young (5.8 ± 1.2 mmol/L) and old (7.3 ± 1.5 mmol/L) mice. We also saw the differences in the groups of old mice on the chow diet (7.3 ± 1.5 mmol/L) and HFD (11.7 ± 1.5 mmol/L).

Thus, at week 1 of the experiment, we performed a glucose tolerance test. Blood glucose reached its maximum at around minute 15. It amounted to 12.35 mmol/L in the chow diet group and 14.2 mmol/L in the experimental group (HFD). At minute 30, the glucose level was lower than at minute 15. By minute 120, it reached values close to the initial level.

**Figure 4:**
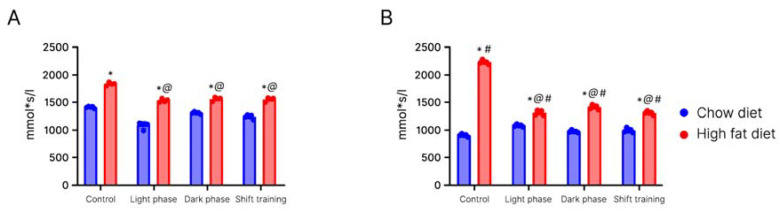
Area under the curve (diagram of blood glucose—week 16). Panel (**A**)—young mice; Panel (**B**)—old mice; data are presented as the mean ± error of the mean. *—Significantly different (*p* < 0.05) from chow diet. #—Significantly different (*p* < 0.05) from young mice. @—Significantly different (*p* < 0.05) from control.

By week 4 of the experiment, the degree of glucose uptake decreased in the experimental group of aged mice. Fifteen minutes after the carbohydrate loading, the blood glucose level in mice of the experimental group reached its maximum—123% of the initial value. After 30 min, the blood glucose began to decline to 93% of the original value. In the chow diet group, the glucose index reached its maximum by minute 15. By minute 120, it was 12% more than the initial value, in contrast with the glucose index at minute 120 in the experimental group, which was 22% of the basal level. In young mice, this effect was registered only by week 12 (Figure 4A).

By week 16 of the experiment, glucose uptake decreased in all groups of mice, but in the old group it was much more pronounced (Figure 4B).

At week 16 of the experiment, the HOMA-IR was 3.62 ± 0.48 in the chow diet group. In the experimental group (HFD), it was 19.85 ± 3.07 (*p* < 0.001). Typically, metabolic disorders are characterized by insulin resistance. As the blood glucose concentration continues to rise, more insulin is released. There were no significant differences between young and old mice. Moreover, no differences were found between different training regimens.

**Figure 5:**
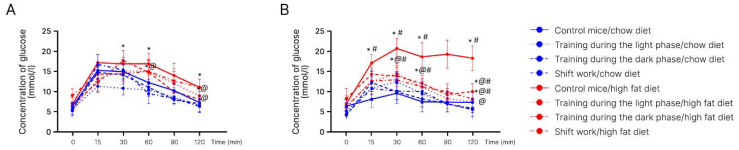
Glucose tolerance test, week 16 (mmol/L). (**A**)—Young mice; (**B**)—old mice. *—Significantly different (*p* < 0.05) from chow diet. #—Significantly different (*p* < 0.05) from young mice. @—Significantly different (*p* < 0.05) from control.

By week 16, the rate of glucose uptake in non-exercised mice from the experimental group increased compared to non-exercised high-fat-fed mice. At 60 min, blood glucose in the exercised mice decreased to 14.46 mmol/L, while the glucose concentration in the non-exercised group remained at 17 mmol/L. By 120 min, in mice subjected to exercise, the blood glucose approached the initial value (6.92 mmol/L) and amounted to 8.35 mmol/L. In the non-exercised group, this difference was 45% (Figure 5A,B).

The hypoglycemic phase indirectly reflects the rate of insulin production and tissue sensitivity to this hormone. The prolongation of this phase is characteristic of metabolic disorders, which was observed in mice of the experimental group in this study.

In chow-fed mice, the maximum rise in glucose concentration was observed at 30 min. It amounted to 15.46 mmol/L (137% of the basal level). Then, the level of blood glucose began to decrease (hypoglycemic phase). By the end of the second hour of observation (by 120 min), it approached the initial level in the chow-fed group (7.94 mmol/L). In mice subjected to exercise, the hypoglycemic phase began even earlier—at 15 min. The glucose level started to decrease and by 120 min dropped to 7.11 mmol/L (17% of the basal level).

We did not find a significant difference in the rate of glucose uptake between the groups subjected to forced running in different phases of the day.

**Figure 6:**
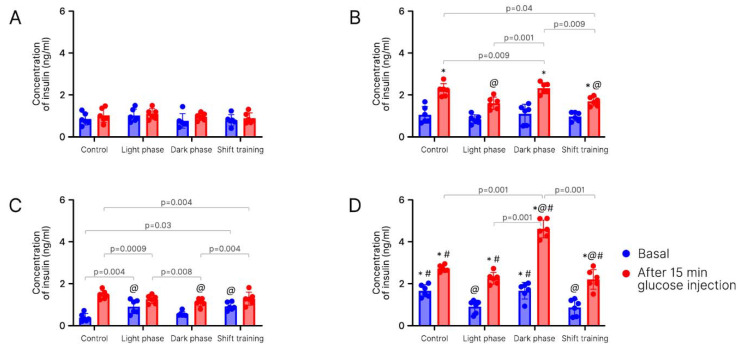
Concentration of insulin in mice blood plasma after 16 weeks (ng/mL). Panel (**A**,**B**)—young mice; Panel (**C**,**D**)—old mice; (**A**,**C**)—chow diet; (**B**,**D**)—high-fat diet. Data are presented as the mean ± error of the mean. *—Significantly different (*p* < 0.05) from chow diet. #—Significantly different (*p* < 0.05) from young mice. @—Significantly different (*p* < 0.05) from control.

Insulin levels in blood plasma. Change in insulin levels in young mice. Figure 6 presents data on changes in insulin levels. There were significant differences only in the high-fat diet groups, only after glucose injection (Figure 6A,B). In the control subgroup, significant differences were observed in the subgroups that exercised in the evening (*p* = 0.009) (dark phase) and performed shift training (*p* = 0.04). The differences were also noticed between the light phase group and the dark phase group (*p* = 0.001) and between the dark phase group and shift training group (*p* = 0.009).

We observed the differences between the chow-fed (1.0 ± 0.3 ng/mL) and high-fat-fed mice (2.3 ± 0.3 ng/mL) in the control subgroup. We also saw the differences between the chow-fed (1.0 ± 0.2 ng/mL) and high-fat-fed mice (2.3 ± 0.3 ng/mL) in the dark phase subgroup. Moreover, we found the differences between the chow-fed (0.9 ± 0.3 ng/mL) and high-fat-fed mice (1.7 ± 0.2 ng/mL) in the shift training subgroup.

In the HFD group, differences were observed between the control subgroup (2.3 ± 0.3 ng/mL) and subgroups of light phase training (1.6 ± 0.3 ng/mL) and shift training (1.7 ± 0.3 ng/mL).

Changes in insulin levels in old mice. Figure 6 presents the significant differences in the control subgroup (0.4 ± 0.2 ng/mL) of the chow-fed group before the glucose injection and in the subgroups of light phase exercise (0.9 ± 0.3 ng/mL) and shift training (0.9 ± 0.2 ng/mL). Moreover, some differences were observed in the control subgroup (1.5 ± 0.2 ng/mL) and dark phase subgroup (1.1 ± 0.2 ng/mL) in the chow-fed group after the glucose injection in the control subgroup (Figure 6C,D).

In the HFD group before the glucose injection, significant differences were observed in the control subgroup (1.6 ± 0.3 ng/mL), light phase subgroup (0.9 ± 0.3 ng/mL), and shift training subgroup (0.9 ± 0.3 ng/mL). After glucose administration in the high-fat-fed group, the control subgroup (2.7 ± 0.1 ng/mL) differed from the dark phase (4.5 ± 0.4 ng/mL) and shift training (2.2 ± 0.2 ng/mL) subgroups.

In the dark phase training group before glucose injection, there were differences between the chow-fed (0.6 ± 0.1 ng/mL) and high-fat-fed mice (1.6 ± 0.4 ng/mL). Moreover, in the control subgroup before glucose injection, there were differences between the chow-fed group (0.4 ± 0.2 ng/mL) and high-fat-fed group (1.6 ± 0.3 ng/mL).

After glucose injection, we saw the differences between the chow diet group (1.5 ± 0.2 ng/mL) and HFD (2.7 ± 0.1 ng/mL) in the control subgroup. The chow-fed mice (1.3 ± 0.2 ng/mL) and high-fat-fed mice (2.2 ± 0.3 ng/mL) demonstrated differences in the light phase subgroup. There were also differences between the chow-fed (1.1 ± 0.2 ng/mL) and high-fat-fed mice (4.5 ± 0.4 ng/mL) in the dark phase training subgroup. The differences between the chow-fed (1.3 ± 0.3 ng/mL) and the high-fat-fed mice (2.2 ± 0.2 ng/mL) were also observed in the shift training subgroup.

Before glucose injection, there were statistically significant differences in the control subgroup exposed to light phase training (*p* = 0.004) and the shift training subgroup (*p* = 0.03) in the chow diet group.

In the chow-fed group after glucose injection, there were significant differences in the control subgroup in comparison with the light phase training subgroup (*p* = 0.0009) and shift training subgroup (*p* = 0.004). Moreover, in the chow-fed group, the differences were found in the dark phase training subgroup (*p* = 0.008) and shift training subgroup (*p* = 0.004).

In the HFD group after glucose injection, the differences were in the dark phase training subgroup vs. control (*p* < 0.001), light phase training (*p* < 0.001), and shift training subgroup (*p* < 0.001).

Differences between old and young mice. Between young (0.9 ± 0.3 ng/mL) and old (0.4 ± 0.2 ng/mL) mice in the chow diet group, a statistically significant difference was present only before the glucose injection in the control subgroup (Figure 6D).

As for young and old mice in the HFD group, before the glucose injection there were differences in the control subgroups (1.1 ± 0.4 ng/mL) (1.6 ± 0.3 ng/mL) and in the dark phase subgroup (1.1 ± 0.5 ng/mL) (1.6 ± 0.4 ng/mL).

We noticed some differences between young and old mice in the HFD group in all subgroups after glucose injection: control (2.3 ± 0.3 ng/mL) (2.7 ± 0.1 ng/mL), light phase training (1.6 ± 0.3 ng/mL) (2.2 ± 0.3 ng/mL), dark phase training (2.3 ± 0.3 ng/mL) (4.5 ± 0.4 ng/mL), and shift training (1.7 ± 0.2 ng/mL) (2.2 ± 0.2 ng/mL). The level of insulin in the group of old mice was significantly higher in all groups compared with the young ones.

In old mice, basal insulin levels were lower than in young mice in the chow diet group and higher than in the high-fat diet group. The effect of glucose injection was somewhat more pronounced in the group of aged mice, especially in the subgroups subjected to forced running. The highest level of insulin was observed in the dark phase training group.

Glut-4 content in muscle tissue. Glut-4 is the main glucose transporter in muscle cells.

**Figure 7:**
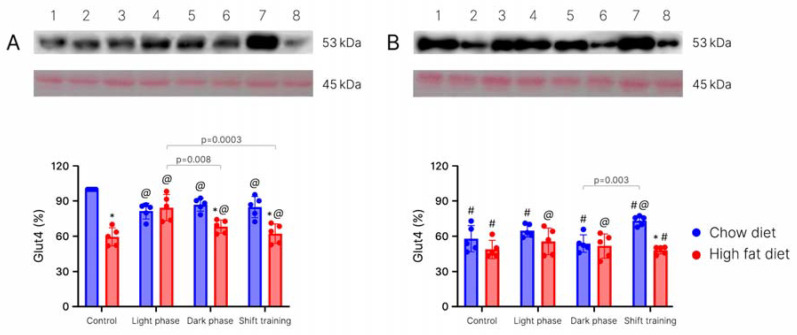
Content of Glut-4 in mice muscle tissue after 16 weeks (% vs. control). Panel (**A**)—young mice; Panel (**B**)—old mice; 1—control/chow diet; 2—control/high-fat diet; 3—light phase/chow diet; 4—light phase/high-fat diet; 5—dark phase/chow diet; 6—dark phase/high-fat diet; 7—shift training/chow diet; 8—shift training/high-fat diet. The data are presented as the mean ± error of the mean. The Ponceau staining is presented as controls for Western blot loading. *—Significantly different (*p* < 0.05) from chow diet. #—Significantly different (*p* < 0.05) from young mice. @—Significantly different (*p* < 0.05) from control.

Changes in Glut-4 content in young mice. In the group of young mice of the chow diet group, all subgroups that were exposed to physical activity differed from the control group (100%) (*p* < 0.05) (Figure 7A). In all cases, we noticed a decrease in the Glut-4 concentration in muscle tissue. The greatest difference was in the light phase training subgroup (81.5 ± 6.8%).

In the group of young mice in the HFD group, the level of Glut-4 in muscle tissue, on the contrary, increased in all exercised subgroups relative to the control group (59.3 ± 7.8%). The greatest increase in the content was observed in the light phase training subgroup (84.3 ± 11.3%) (*p* < 0.05).

Moreover, we noticed the differences (*p* = 0.008) between the light phase training subgroup (84.3 ± 11.3%) and dark phase training subgroup (68.1 ± 5.7%). The Glut-4 content in the subgroup of exercised mice (62.1 ± 8.3%) also differed significantly (*p* = 0.0003) relative to the subgroup with light phase training (84.3 ± 11.3%).

We found out that there were differences between groups with different types of nutrition in the control group, as well as in the shift training group compared to the dark phase group (*p* < 0.05). There was a decrease in the level of Glut-4 in muscle tissue in high-fat-fed mice.

Changes in Glut-4 content in old mice. In chow-fed old mice (*p* < 0.05), we observed the differences in the shift training subgroup (71.3 ± 4.1%) in comparison with the control group (56.4 ± 10.9%) (Figure 7B). Moreover, the content of Glut-4 in this subgroup was higher than in the dark phase subgroup (52.3 ± 7.2%) (*p* = 0.003).

In high-fat-fed old mice, the differences (*p* < 0.05) were noticed in two subgroups: the light phase (54.1 ± 11.0%) and dark phase (50.4 ± 9.9%). The value in the control subgroup was 47.6 ± 7.4%.

The differences between groups with different types of nutrition were observed only in the group with shift training (*p* < 0.05). The content of Glut-4 in muscle tissue in the HFD mice was 34% lower than in chow-fed mice.

Differences between young and old mice. The Glut-4 content in muscle tissue was significantly lower in all subgroups of chow-fed old mice than in young mice. In the aged subgroup with shift training, Glut-4 was lower by 16%. In the aged light phase and dark phase training subgroups, it was lower by 22.5% and 40%, respectively. In the old control group, it was by 44% lower than in young mice (*p* < 0.05).

The differences (*p* < 0.05) between the HFD groups of young and old mice were observed only in two subgroups. In young mice of the control subgroup (59.3 ± 7.8%), the values were higher than in old mice (47.6± 7.4%). We noticed the same trend in the shift training subgroup.

In the HFD group, the Glut-4 content in muscle tissue was significantly lower than in the chow diet group (Figure 7A). Forced exercise led to a significant increase in the concentration of Glut-4 in muscle tissue in young animals on the high-fat diet. At the same time, the greatest increase was observed in the light phase group.

In the group of old animals, the content of Glut-4 in muscle tissue was significantly lower than in young animals on the chow diet (Figure 7B). Accordingly, high-fat-fed older mice showed lower levels of Glut-4. Forced physical activity led to its slight increase. There was no difference between the subgroups performing physical exercise at different times of the day.

**Figure 8:**
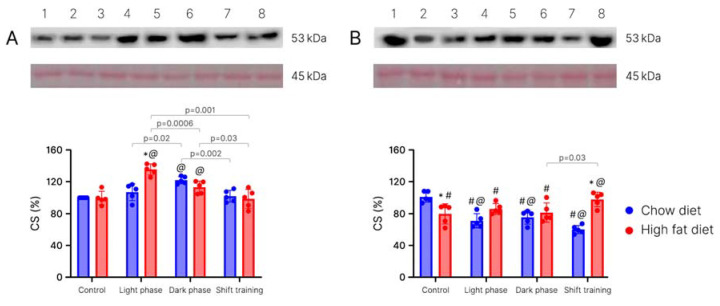
Content of CS in mice muscle tissue after 16 weeks (% vs. control). Panel (**A**)—young mice; Panel (**B**)—old mice; 1—control/chow diet; 2—control/high-fat diet; 3—light phase/chow diet; 4—light phase/high-fat diet; 5—dark phase/chow diet; 6—dark phase/high-fat diet; 7—shift training/chow diet; 8—shift training/high-fat diet. The data are presented as the mean ± error of the mean. The Ponceau staining is presented as controls for Western blot loading. *—Significantly different (*p* < 0.05) from chow diet. #—Significantly different (*p* < 0.05) from young mice. @—Significantly different (*p* < 0.05) from control.

Content of citrate synthase (CS) in muscle tissue. Changes in CS content in young mice. The content of CS in muscle tissue in the chow diet group of young mice differed between the control group (100%) and dark phase training group (122.2 ± 4.6%) (*p* < 0.05) (Figure 8A).

Furthermore, we observed some differences between the exercised subgroups within the chow diet group of young mice. In the dark phase subgroup, the CS content was 14% higher than in the light phase subgroup and 19% higher than in the shift training subgroup (*p* = 0.002).

In the HFD group, there were differences (*p* < 0.05) both in the dark phase subgroup (113.3 ± 7.7%) and in the light phase subgroup (135.8 ± 7.0%) as compared to the control group (99.5 ± 8.6%). The CS content in the light phase subgroup (135.8 ± 7.0%) was higher than in the dark phase subgroup (113.3 ± 7.7%) (*p* = 0.0006). We observed the same trend relative to the shift training subgroup (98.6 ± 12%): the CS content was lower by 27% (*p* = 0.001).

Different types of nutrition influenced the groups subjected to light phase exercise (*p* < 0.05). The CS content in the chow diet group (107.0 ± 10.6%) increased by 26% in comparison with the HFD group (135.8 ± 7.0%).

Changes in CS content in old mice. In the chow-fed old mice, differences (*p* < 0.05) were observed in all three subgroups exposed to physical exercise in comparison with the control group (98.0 ± 6.5%). We noticed the lowest concentration in the shift training subgroup: 58.2 ± 5.0% (Figure 8B).

In the high-fat diet group, there were differences in the shift training subgroup (94.9 ± 8.8%) compared to the dark phase subgroup (78.8 ± 11.7%) (*p* = 0.03).

Between the groups with different types of nutrition, the differences were observed in the shift training subgroup (*p* < 0.05). The CS content in the chow diet group (58.2 ± 5.0%) was 38% lower than in the HFD group (94.9 ± 8.8%). In the control group, on the contrary, the content of CS in the chow diet group was 21% higher than in the HFD group.

Differences between young and old mice. The differences between old and young mice in the chow diet group were observed in all groups exposed to physical activity (*p* < 0.05). In old mice, the CS content was lower. In the light phase training group, the content was 35% less than in young mice. In the dark phase training group, it was 40% less. In the shift training group, it was 42% less (Figure 8B).

In the HFD groups, we saw the differences in the light and dark phase subgroups, as well as in the control subgroup (*p* < 0.05). The level of CS in old mice was also lower relative to the group of young mice. It was 22% lower in the control group, 39% lower in the light phase subgroup, and 30% lower in the dark phase subgroup than in the same subgroups of young mice.

The high-fat diet in young mice did not affect the CS content in muscle tissue. Forced physical activity led to an increase in this indicator. To the greatest extent, this was manifested in the subgroup exposed to loads during the daytime (Figure 8A).

In aged mice, the HFD contributed to a decrease in the CS concentration in the control group. Physical activity did not significantly affect this indicator. In all aged subgroups, it remained lower than in young animals (Figure 8B).

## 3. Discussion

The results obtained indicate that a high-fat diet in mice leads to hyperglycemia, reduced glucose tolerance, and hyperinsulinemia. It causes body weight gain and obesity: the body weight in the experimental group is more than 25% higher than in the control group. All these testify to the adequacy of the developed experimental model of metabolic disorders. The criteria for the adequacy of the model can be considered as follows: the dynamics of body weight; hyperglycemia; results of a glucose tolerance test, including the area under the glucose concentration curve in the glucose tolerance test (AUC) [36]. These results were also confirmed in studies on mice of four different strains. Li et al. found that an HFD induces obesity in C57BL/6J and BALB/c mice and increases their body weight [37]. The body weight of HFD mice was higher than that of controls. A similar result was obtained in [38].

Obese mice generally exhibited a slower and weaker ability to metabolize elevated blood glucose during a high carbohydrate loading [39]. It was clear that exposure to a high-fat diet impairs the mice’s ability to control glucose levels.

An increase in body weight and deterioration in glucose uptake may be the result of the formation of insulin resistance. Insulin resistance is one of the hallmarks of obesity, which includes mild inflammation and blockage of several insulin signaling pathways [40].

In addition, in middle-aged mice, the increase in the ratio of fat to body weight and the accumulation of lipids in the liver are greater than in young mice [41].

At the same time, the high concentration of insulin in the blood of the animals of the experimental group indicates that disorders develop only in muscle tissue, while the sensitivity of β-cells of the pancreas to glucose is preserved. This allows us to conclude that the developed model is adequate. We modeled disorders of the muscle tissue but not the pancreas. Perhaps, the formation of resistance to glucose on the part of β-cells of the pancreas takes more time. 

Forced physical activity in the form of daily treadmill running has a number of pronounced effects on metabolism in mice with metabolic disorders. Firstly, we observed body weight loss, which manifested itself to a greater extent in young animals. We found out that weight loss depends on the phase of the day physical activity is performed. Moreover, physical activity is accompanied by an increase in the rate of glucose uptake and a significant increase in insulin concentration. The effects are pronounced in aged mice.

All of the above indicate that regular physical activity improves carbohydrate metabolism. It also results in the involution of changes characteristic of metabolic disorders. Moreover, the mechanism of these changes may be related to the change in Glut-4. The HFD in mice is accompanied by a decrease in the content of Glut-4 in muscle tissue. This has also been confirmed in studies that revealed that the expression of Glut-4 was lower in the HFD group of mice [42]. An increase in the content of Glut-4 against the background of physical activity has been confirmed in a number of studies [43,44].

During exercise, the Glut-4 production increases, which improves insulin sensitivity. Improved insulin sensitivity, in turn, increases glucose uptake and finally improves glycemic control [43]. This improvement in insulin sensitivity contributes to the improvement of glycemic control towards the normal range [44]. Bird and Hawley found that regular exercise reduces the risk of insulin resistance and also improves insulin sensitivity [45].

Moreover, in the studies of Wang et al., the IRS-1/Akt/Glut-4 signaling pathway was reduced in obese mice and activated during exercise. This resulted in an increased uptake and accumulation of glucose in skeletal muscle [42].

However, in our study, we discovered that in the group of chow-fed young mice, there was a multidirectional reaction in terms of the content of Glut-4 against the background of physical activity. Glut-4 decreased in the group of chow-fed mice and increased in the group of high-fat-fed mice. The greatest increase was in the light phase subgroup.

The study showed that Glut-4 has a short half-life, and its expression can change rapidly. There is some controversy regarding how long elevated Glut-4 levels persist after exercise [29].

We saw Glut-4 increase in old mice during exercise. Thus, we may suggest that increased Glut-4 normalizes carbohydrate metabolism in aged mice during exercise.

Many authors associate the mechanisms of the described effects of physical activity with the endocrine function of skeletal muscles [46,47,48]. The relationship between the contractile activity of skeletal muscles and transcription processes has been proven [49,50,51,52,53,54,55]. At the same time, changes in the transmembrane gradients of sodium and potassium ions might play an important role in these processes [56].

Myokines entering the systemic circulation can have various effects on energy metabolism. Our previous publication showed that treadmill training caused multidirectional changes in the concentration of myokines in muscle tissue [57]. The content of IL-6 has changed most profoundly. These changes were observed in all groups of animals. The changes depended on the temporal scheme of training to the greatest extent. The effect of exercise on the content of IL-15 in skeletal muscle tissue was observed mainly in 48-week-old mice. In 20-week-old animals, exercise increased LIF concentration in muscle tissue during dark phase training or shift training. In the HFD group, this effect was significantly more pronounced. The content of CXCL1 did not change in almost all groups of animals exposed to exercise.

Other studies have identified the effects of certain myokines on energy metabolism. In particular, IL-6 is a cytokine that can be released in skeletal muscle during exercise. After binding to its receptor, IL-6 myokine activates the PI3K-Akt pathway. One consequence of this activation is the potentiation of insulin signaling, which increases insulin sensitivity. IL-6 increases the mobilization of Glut-4 vesicles to the periphery of the muscle cell and increases the transport of glucose into the cell, as well as the synthesis of glycogen. Thus, this myokine may be the factor that changes energy metabolism against the background of physical activity [58].

Our study showed that CS increases in young mice during exercise. This effect was also confirmed in a study on rats [59]. Older mice on the high-fat diet also had an increase in CS. These effects are confirmed in studies that show a decrease in CS activity against the background of a decrease in motor activity [60]. However, normal-fed old mice showed a decrease in CS levels. MacInnis et. al. has proven that the CS activity increases only after high-intensity exercise [61]. In addition, some studies have noted impaired mitochondrial function and adaptation to physical activity in aged animals [62]. 

Physical activity is important for finding new ways to correct metabolic disorders. Taking into account circadian rhythms and age-related characteristics, it is a promising way to influence metabolic processes both at the cellular and systemic levels.

## 4. Materials and Methods

Male mice of the C57bl/6 line were obtained from the vivarium of E.D. Goldberg Research Institute of Pharmacology and Regenerative Medicine. Animal keeping mode: day/night: 12/12 h, daylight hours began at 6:00 am, free access to food and water, room temperature 24 °C.

This study was conducted in accordance with the principles of the Basel Declaration and approved by the Commission on Bioethics of the Biological Institute of Tomsk State University (protocol No. 32, dated 2 December 2019).

**Figure 9:**
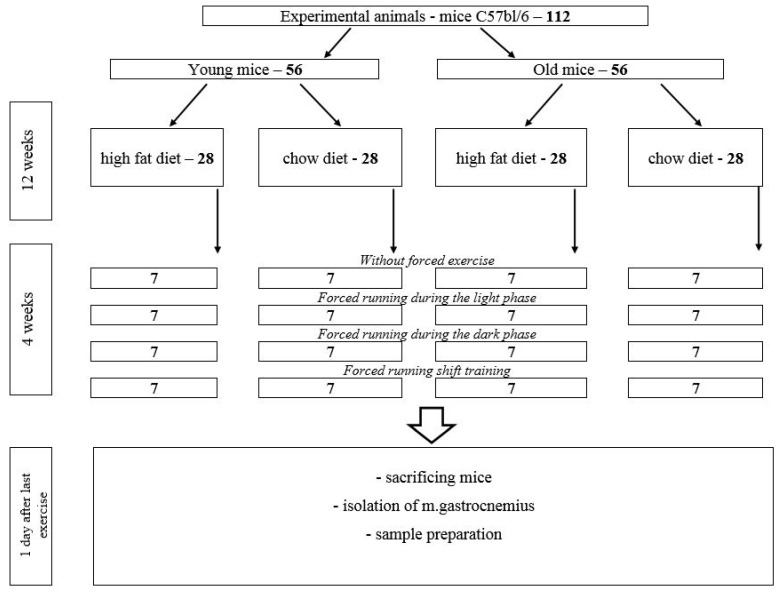
Experiment design.

The scheme of the experiment is shown in Figure 9. 

In total, 112 mice were assigned into two groups of different ages. The young group consisted of 56 mice included in the experiment at the age of 4 weeks; the old group consisted of 56 mice included in the experiment at the age of 32 weeks.

The experiment lasted 16 weeks. Until week 12, mice of each age group were divided into 2 subgroups (Figure 9):

- Animals on a high-fat diet (HFD)—28 mice (experimental group).
- Animals on a chow diet—28 mice (control group).

We developed an HFD specifically for the experiment to form a model of metabolic disorders in mice. Mice were on an HFD for 12 weeks. The composition and energy value of the feed are described in detail in our previous work [63].

The control group ate food for laboratory animals, Prokorm (CJSC Biopro, Novosibirsk): wheat, barley, bran, corn gluten, fish meal, protein feed mixture, sunflower oil, and soybean meal. The energy content made up 3000 kcal/kg, including 18% fats.

The experimental group of mice ate a high-calorie food. The food for the experimental group was prepared on the basis of Prokorm (50%) with added animal (pork fat) (20%) and vegetable (sunflower oil) (10%) fat, sugar (15%), and milk powder (5%). The energy content made up 5100 kcal/kg including fats, which accounted for 59% of calories (Table 1).

Our preliminary study [64] showed that the HFD leads to body weight gain, obesity, hyperglycemia, decreased glucose tolerance, and hyperinsulinemia. Therefore, the developed experimental model of type 2 diabetes is valid and reliable. At week 12, both experimental and control groups of animals were further divided into four subgroups each according to the load and time of physical exercise (Figure 1).

Subgroup 1 consisted of 7 animals that were not exposed to forced treadmill running (the control group).

Subgroup 2 included 7 animals that were exposed to treadmill running during daylight hours (from 8:00 to 10:00) (the light phase group).

Subgroup 3 consisted of 7 animals that were exposed to treadmill running in the evening hours (from 19:00 to 21:00) (the dark phase group).

Subgroup 4 included 7 animals that had an alternated time of forced treadmill running (shift training): weeks 1 and 3 the animals exercised in the evening hours (from 19:00 to 21:00), and weeks 2 and 4 they exercised in the daytime (from 8:00 to 10:00) (the shift training group). To standardize the exercises, we used a BMELAB SID-TM10 treadmill for mice [64].

Forced treadmill running was carried out 6 times a week for 4 weeks. The duration of the load gradually increased during the first 6 days from 10 to 60 min (an increase of 10 min per day) and did not change over the next 3 weeks. Every week we changed the angle of the treadmill elevation (from 0 to 10o) and rotation speed (from 15 to 18 m/min). Once a week, mice were not exposed to physical exercise (day 7).

### 4.1. Research Methods

Body weight was measured using laboratory scales. Each individual was measured separately. We took measurements 11 times over 16 weeks. 

Animals were sacrificed by decapitation 24 h after the last load. We dissected M. gastrocnemius from both hind limbs. We cleaned muscle tissue from connective and adipose tissue. After that, we put the collected samples in a freezer and stored them at −80 °C. Then, we analyzed the samples with electrophoresis and Western blotting.

#### 4.1.1. Glucose Tolerance Test

Veins of the mouse tail were punctured for blood sampling. We measured blood glucose concentration using a portable glucometer PKG-02.4 Satellite Plus (ELTA, Moscow, Russia). 

To test the glucose tolerance, all mice were fasted 4 h before the test. They had free access to water. In the morning, we weighed the animals and measured fasting blood glucose levels (0 min). The animals were then intraperitoneally injected with a solution of 40% glucose (2 g/kg body weight) (carbohydrate loading). We measured blood glucose at 15, 30, 60, and 120 min after the carbohydrate loading. We also evaluated the maximum concentration reached, the time to peak, and the time to return to baseline.

#### 4.1.2. Measurement of Insulin Concentration in Blood Plasma

We punctured the tail vein for blood sampling and collected blood in capillary tubes Microvette Sarstedt (Sarstedt, Nümbrecht, Germany) 200 µL with K3EDTA.

Samples were centrifuged immediately after blood sampling for 6 min at 10,000 rpm at 4Co using a Microfuge 16 laboratory centrifuge with an FX 241.5P rotor (Beckman Coulter, Chaska, MN, USA). Plasma was stored frozen at −80 °C for no more than a month. We determined insulin concentrations in the blood plasma of mice by enzyme immunoassay using the ultra-sensitive mouse insulin ELISA Kit (CrystalChem, Elk Grove Village, IL, USA). We used 12 × 8 plates for the analysis. The total number of flat-bottomed wells was 96. All samples were added in duplicates to wells in the plates. Then, the samples were diluted according to the instructions and incubated in a thermoshaker for plates PST-60HL (Biosan, Riga, Latvia). After that, we carried out the washing procedure using an Anthos Fluido 2 washing device (Biochrom, Cambridge, UK).

The optical density of the samples was measured using an Anthos 2010 microplate spectrophotometer with filters (400–750 nm) and the ADAP+ program (Biochrom, UK). The optical density of the samples was calculated at a wavelength of 450 nm, and the reference wavelength was 620 nm. Serial dilutions of highly concentrated protein solutions included in the kits were used to prepare the standards. 

#### 4.1.3. Determination of the Citrate Synthase in Muscle Tissue

Following the protocol and instructions, we performed muscle tissue homogenization for further Western blotting. We used Anti-Citrate synthetase antibody (ab96600) (Great Britain).

For Western blot analysis, we performed polyacrylamide gel electrophoresis under denaturing conditions using an electrophoresis system (electrophoresis cell (Mini-PROTEAN Tetra Bio-Rad, Woonsocket, RI, USA), power supply (PowerPacBasic, BioRad, Hercules, CA, USA)). According to the method described by Laemmli (1970), we used 5% concentrating and 7% separating gels. 

#### 4.1.4. Determination of the Glut-4 in Muscle Tissue

We measured muscle Glut-4 with Western blotting using CBL243 Anti-Glucose Transporter Glut-4 Antibody, Sigma-Aldrich (USA). Western blot data are presented in relative units. We added 10 μg of protein to each well. For total protein concentration analysis, we used the Bradford micro method (reagent Thermo Fisher Scientific, Cat. No. 20279).

Target proteins were detected by overnight incubation at 4 °C in 5% milk powder in TBSt at a 1:1000 dilution with rabbit polyclonal Glut-4 antibodies (Cat. No. CBL243, Sigma-Aldrich, St Saint Louis, MO, USA). Then, we incubated the samples with HRP-conjugated secondary antibodies (anti-mouse Cat. No. 1706516, anti-rabbit Cat. No. 1706515, BioRad, Hercules, CA, USA) for 1 h at room temperature in 5% dry milk in TBSt.

We visualized antigen–antibody complexes using an ECL kit (SuperSignal West Dura, Thermo Scientific, Waltham, MA, USA) and a documentation system (ChemiDoc-It 2, Ultra-Violet Products, London, UK). We used Ponceau staining as controls for Western blot loading. Control values were taken as 100%. Densitometric analysis was carried out using Image G software (https://imagej.net/).

#### 4.1.5. Statistical Analysis

All analyses were performed with GraphPad Prism 9.3.1, and datasets were assessed for normality and group variance prior to statistical testing. Values are expressed as mean ± SEM, unless otherwise stated. Post-test corrections were used every time multiple comparisons were performed. All data had an abnormal distribution of the feature. Two-way ANOVA with Tukey’s multiple comparison test and Holm–Sidak correction was used. AUC calculation was performed with GraphPad Prism 9.3.1. Statistical processing of the results was performed using the GraphPad Prism application package. 

## 5. Conclusions

The results obtained allow us to draw several important conclusions.

Firstly, daily treadmill running has different effects on different age groups of mice with metabolic disorders. In young-age animals, forced running has a more pronounced effect on body weight, while in older animals it influences the rate of glucose uptake.

Secondly, the effects of physical activity depend on the time of day. The greater effect is observed when performing shift training or exercise during the time when animals are passive (light phase).

In young mice, light phase training had a significant effect on increasing the content of Glut-4 in muscle tissue. In aged mice, shift training caused an increase in the level of Glut-4 in muscle tissue.

In the group of aged mice, a lower CS level was noticed in all groups in comparison with young mice. It should also be noted that we observed that CS increased during exercise in the group of young mice, especially during light phase training. The CS decreased in aged chow-fed mice and increased in the high-fat-fed group.

The results obtained reveal a promising way of influencing metabolic processes both at the cellular and systemic levels. 

### Limitations

Our studies have a few limitations. A limitation of this study is the use of the minimum allowed number of mice per group. We did not calculate accurately animal numbers with respect to changes in amplitude parameters. The high-fat diet we used is not certified, but it was tested in our previous studies. The use of this diet led to the development of metabolic disorders in mice. A limitation of our work is the lack of objective control of the amount of food consumed by the mice. We did not isolate the membrane proteins fraction and analyzed Glut-4 content. The Glut-4 product does not show real Glut-4 or glucose uptake activity. In this study, we researched Glut-4 products against the background of various conditions (training at different times of the day, different ages, different diets). A limitation is the fact that we did not determine FBG and HbA1c, which are relevant parameters for the diabetes model. However, our model is not diabetes in the truest sense. The duration of the experiment, in our opinion, did not allow us to achieve a significant change in these parameters.

## Figures and Tables

**Table 1:** Characteristics of diets for the experimental (high-fat diet) and control groups (chow diet).

Characteristics	High Fat Diet	Chow Diet
Caloric content, kcal/kg	5100	3000
Including % of calories from fat	59%	2.5%
Composition		
Fats	33.3%	6%
Including animal fats	25%	-
Carbohydrates	17%	3.6%
Protein	13%	23.9%
Lysine	0.8%	1.5%
Methionine + Cysteine	0.5%	0.9%
Macronutrients		
Calcium	0.9%	1%
Phosphorus	0.7%	0.8%
Sodium chloride	0.24%	0.34%
Vitamins and minerals	+	+
Antioxidant, amino acids	+	+

## Data Availability

Data is contained within the article.
